# Promising outcomes of R-CHOP therapy in pediatric nodular lymphocyte-predominant Hodgkin lymphoma: perspectives from a rare subtype

**DOI:** 10.3389/fonc.2026.1740063

**Published:** 2026-03-16

**Authors:** Nesreen Ali, Eman Khorshed, Mohamed S. Zaghloul, Maha Mehsen, Sarah Badawy, Walaa Elsayed

**Affiliations:** 1 Department of Pediatric Oncology, National Cancer Institute, Cairo University, Cairo, Egypt; 2 Department of Pediatric Oncology, Children’s Cancer Hospital Egypt 57357, Cairo, Egypt; 3 Department of Pathology, National Cancer Institute, Cairo University, Cairo, Egypt; 4 Department of Pathology, Children’s Cancer Hospital Egypt 57357, Cairo, Egypt; 5 Department of Radiation Oncology, National Cancer Institute, Cairo University, Cairo, Egypt; 6 Department of Radiation Oncology, Children’s Cancer Hospital Egypt 57357, Cairo, Egypt; 7 Department of Nuclear Medicine, National Cancer Institute, Cairo University, Cairo, Egypt; 8 Department of Nuclear Medicine, Children’s Cancer Hospital Egypt, Cairo, Egypt; 9 Department of Clinical Research, Children’s Cancer Hospital Egypt 57357, Cairo, Egypt

**Keywords:** event free survival, nodular lymphocyte predominant Hodgkin lymphoma, outcomes, pediatric, R-CHOP

## Abstract

**Background:**

Nodular lymphocyte predominant Hodgkin lymphoma (NLPHL) is a rare sub-type of Hodgkin lymphoma (HL). Due to its rarity, the standard of care for these patients remains poorly defined. Our cohort aimed to evaluate the outcomes of pediatric NLPHL patients treated with the R-CHOP regimen throughout 8 years’ single center experience.

**Methods:**

A retrospective cohort study including pediatric patients aged <18 years diagnosed with NLPHL and treated at the Children Cancer Hospital Egypt, between July 2014 and June 2022.

**Results:**

In total, 66 patients were included. Twelve patients (18%) with stage IA underwent surgical excision alone. Four of these patients experienced disease progression but achieved a complete response after receiving R-CHOP. Fifty-four patients received R-CHOP as first-line therapy, with early-stage disease received 3–4 cycles, while those with advanced-stage disease received 6 cycles. For this group, the 5-year overall (OS) and event free survival (EFS) were 100% and 91.5%, respectively. The 5-year EFS was not significantly affected by the variant histology pattern among patients received R-CHOP. Patients with stage 3 peripheral disease without risk factors demonstrated outcomes comparable to those with early-stage disease and no risk factors, achieving an excellent 5-year EFS rate of 100%. No significant toxicity was reported, except for grade 3–4 neutropenia.

**Conclusion:**

R-CHOP is an effective and well-tolerated regimen for treating pediatric NLPHL, demonstrating high OS and EFS rates. Patients with early stage disease achieved particularly favorable outcomes, supporting consideration of treatment de-escalation in this subgroup.

## 1 Introduction

Nodular lymphocyte predominant Hodgkin lymphoma (NLPHL) is a rare subtype of Hodgkin lymphoma (HL), accounting for about 10% of all HL cases (1–3). It is a unique pathological type of B-cell lymphoma that carries a favorable outcome (4, 5), and it has been lately relabeled as nodular lymphocyte-predominant B-cell lymphoma (NLPBL) due to its strongest match to T-cell/histiocyte-rich large B-cell lymphoma (THRLBCL), as evident by gene expression profiling analysis (6). Its propensity to affect peripheral locations, early stage disease, and indolent behavior set it apart from classical HL (cHL) (7). Doxorubicin, bleomycin, vinblastine, and dacarbazine—treatment regimens designed for cHL such as ABVD—have been linked to increased transformation to aggressive B-cell lymphomas of diffuse Large B-cell type (DLBCL) and late relapse rates (1). Since CD20 is universally expressed by the lymphocyte-predominant (LP) cells of NLPHL, treatment with anti-CD20 antibody (rituximab)-containing regimens as rituximab, cyclophosphamide, doxorubicin, vincristine, and prednisone (R-CHOP), may substitute the ABVD regimen (8). Numerous studies conducted by Fanale et al. demonstrated excellent efficacy of R-CHOP in NLPHL patients (9, 10), with an overall response rate (ORR) of 100%, including about 90% complete response (CR), 5-year progression-free survival (PFS) reaching up to 95%, and even 5-year PFS ranging from 86% to 90% for those with advanced-stage disease. Most of the published data on the outcome of R-CHOP in NLPHL are derived from adult populations, with only a limited number of studies addressing pediatric patients. Moreover, the available pediatric reports are scarce and often involve heterogeneous treatment regimens rather than a uniform R-CHOP protocol. Therefore, this study aims to analyze the outcome of R-CHOP specifically in pediatric patients with NLPHL, based on an eight-year single-center experience.

## 2 Patients and methods

### 2.1 Study design

**Figure 1:**
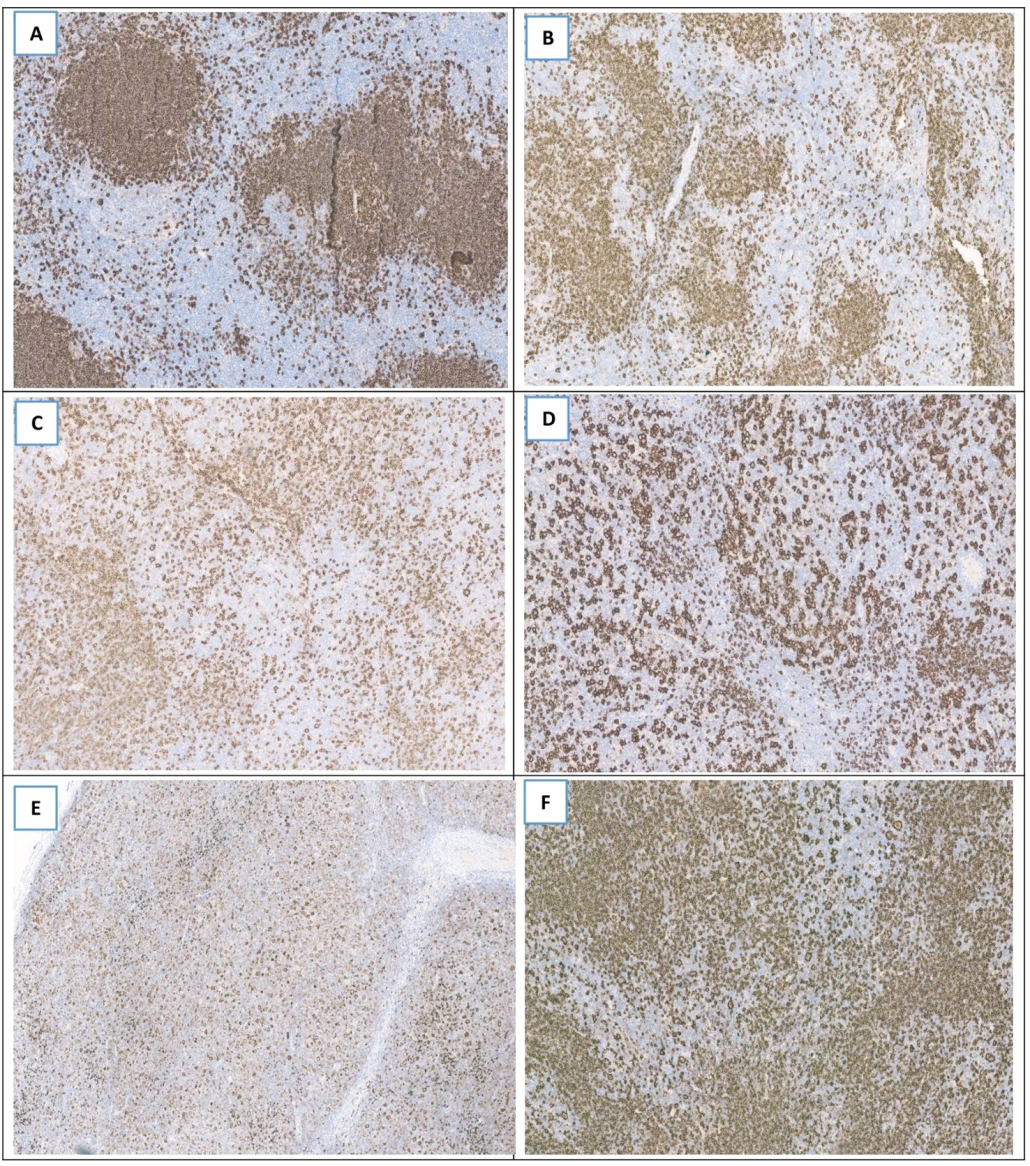
Six different cases of NLPHL with different patterns as highlighted by CD20 immunostaining. **(A)** Typical pattern A showing classical nodular appearance with LP cells detected within the nodules. **(B)** Typical pattern B of serpiginous/interconnected nodular appearance where LP cells are seen within the nodules and some are extranodular. **(C)** Variant pattern C showing nodular pattern with prominent extra-nodular LP cells. **(D)** Variant pattern D characterized by T-cell-rich background with still noted nodular areas. **(E)** Variant pattern E that shows T-cell-rich background without appreciated nodular areas. **(F)** Variant pattern F with predominant B cell rich background.

We included all consecutive eligible pediatric NLPHL patients diagnosed and treated at the Children’s Cancer Hospital Egypt (CCHE) between July 2014 and June 2022 in this retrospective study. Two skilled hemato-pathologists at our center reviewed the cases of eligible patients, who were between the ages of 1 and 18 at the time of diagnosis and had confirmed pathology of NLPHL, in order to rule out other possible diagnoses and confirm this rare disease entity. Fan and colleagues’ criteria were used to classify the tumor growth pattern as either morphologically variant or typical (11, 12) (**Figure 1**). For staging purposes and for early response evaluation following two cycles of R-CHOP, all patients had Fluorodeoxyglucose Positron emission tomography–computed tomography (FDG PET–CT) performed. Late response assessment is done at the end of chemotherapy cycles by either PET-CT or computed tomography (CT) scan. Using the Ann Arbor system, disease staging was established (13). Patients with stage I and II were classified as early stage, while those with stage III and IV were considered advanced stage (5).

Patients were classified according to Ann Arbor stage and extent of surgical resection, which directly determined management at our institution into Stage IA, completely resected, treated with observation only. Stage I–II, not completely resected irrespective of clinical baseline factors received 4 cycles of R-CHOP (only 9 patients of early stages I-IIA unresected received 3 cycles of R-CHOP after 2020 as most of literature recommend to give 3 cycles of less intensive chemotherapy and we updated protocol at that time). Stage III–IV received 6 cycles of R-CHOP. Baseline clinical factors (e.g., B symptoms and bulky disease) were recorded for descriptive purposes and exploratory analyses.

Risk factors included B symptoms, splenic involvement, mediastinal lymphadenopathy, or variant histology. When the largest lymph node measures more than 6 cm in diameter, the condition is referred to as “bulky lymph node disease.” Relapse was categorized as early if it occurred within 24 months after initial diagnosis, or late if it occurred more than 24 months of initial treatment.

Data were extracted from the unified electronic medical record. Patients were identified via the pathology database/EMR using predefined criteria. Clinical, treatment, response, and outcome data were abstracted using a standardized form and cross-checked with pathology, imaging/PET-CT, chemotherapy administration, and follow-up documentation.

### 2.2 Treatment strategy

Patients with stage IA disease who underwent complete excision of one affected lymph node were observed without further medical intervention. Other patients were given the R-CHOP regimen, which included vincristine, doxorubicin, cyclophosphamide, prednisone, and rituximab, in an outpatient setting. Rituximab is administered at a dose of 375 mg/m² intravenous (IV); cyclophosphamide is given at 750 mg/m² IV, followed by doxorubicin at 50 mg/m² IV and vincristine is administered at 1.4 mg/m² (maximum 2 mg) IV, all on day 1. Prednisone is given orally at 100 mg/m²/day (or 40–60 mg/m²/day) from days 1 to 5. A combined modality treatment regimen (R-CHOP+ involved site radiotherapy, ISRTH) was used to treat patients with NLPHL prior to 2016. Thereafter, only Patients who had an inadequate response, as evident by PET-CT positivity, after the 2nd cycle of R-CHOP received ISRTH at a dose of 1980 cGy (11 fractions) at the end of chemotherapy cycles. Relapse was confirmed by a new tissue biopsy. Relapsed patients post R-CHOP received salvage chemotherapy with the RICE regimen (rituximab, ifosfamide, carboplatin, and etoposide) with four to six cycles. Those with negative PET-CT after 2 cycles received consolidation treatment with ISRTH at the end of chemotherapy cycles. Autologous stem cell transplantation (ASCT) was offered to patients demonstrating an inadequate response following the second cycle of salvage R-ICE, as indicated by persistent PET-CT positivity, or in cases of a second relapse. Initial disease characteristics, histological variants of the disease, response after 2 cycles of R-CHOP, reported adverse events and the final outcome were collected from electronic medical records.

### 2.3 Response criteria

Overall response rate (ORR) was defined as the sum of patients achieving complete response (CR) and partial response (PR) following upfront systemic therapy. CR was defined as disappearance of all clinical and radiological evidence of disease, with no residual extra-mediastinal nodal mass larger than 2.0 cm and a negative FDG-PET (Deauville score <4). PR was defined as a reduction of more than 50% in the sum of the products of the perpendicular diameters of target nodal or extra-nodal lesions. Progressive disease (PD): an increase of ≥50% in the product of the perpendicular diameters of any nodal mass, or the appearance of new lesions indicating progression (14).

### 2.4 Assessment of toxicity

Using the Common Terminology Criteria for Adverse Events (CTCAE), version 5, adverse events were grouped. Only grades 3 and 4 of hematologic toxicities were reported, but all grades of hypersensitivity reactions were noted. All forms of infections, fever, and any toxicity that required a change in dosage were also assessed.

### 2.5 Statistics

All statistical analyses were performed using R software, version 4.4.1. The core packages survival (for statistical modeling) and survminer (for visualization) were utilized for time-to-event analyses. Patient characteristics and clinical data were summarized using descriptive statistics. The median and range were used to describe quantitative data, and the means were also given when they were needed. Categorical variables were summarized as frequencies and percentages. Comparisons between groups were conducted using the chi-square test or Fisher’s exact test for categorical variables, with the latter used when expected cell counts were less than five. For continuous variables, the distribution of data was assessed for normality. The independent samples t-test was used for normally distributed data, while the non-parametric Mann-Whitney U test was applied for non-normally distributed data. The primary endpoints were Overall Survival (OS) and Event-Free Survival (EFS). OS was defined as the interval from the date of diagnosis to death from any cause, with surviving patients censored at the date of last follow-up. EFS was defined as the time from the date of achieving remission to the first occurrence of an event, which included disease progression, relapse, or death from any cause. Patients alive and event-free were censored at the date of last follow-up. Survival probabilities were estimated using the Kaplan-Meier method, and differences between survival curves were evaluated using the log-rank test. A two-sided p-value of <0.05 was considered statistically significant for all tests.

## 3 Results

### 3.1 Patient characteristics

The study included 66 pediatric patients diagnosed with NLPHL at the CCHE. Twelve patients (18%) were managed with surveillance after undergoing primary surgical excision and having negative PET-CT results. The R-CHOP regimen was used as systemic therapy for the remaining 54 patients. Fifty patients of the whole cohort were male (75.8%), 35 were older than 10 years (53%) and with peripheral lymphadenopathy in 54 patients (81.8%). Mediastinal lymphadenopathy was observed in only 7 (10.6%) patients. Stage II disease was the most common presentation (40.9%), followed by stage I (36.4%), while only a minority of patients (22.7%) presented with advanced stages. B symptoms were observed in 8 patients (12.1%), and splenic involvement in 4 patients (6.1%). Bulky disease was identified in 7 patients (10.6%). Most patients had supra-diaphragmatic lymphadenopathy (62.1%), whereas 14 patients (21.2%) exhibited both supra- and infra-diaphragmatic involvement. Half of the patients (33) showed classic nodular or nodular interconnected/serpiginous histology (types A and B). The remaining half exhibited a variety of histological variants (types C, D, E, and F). The demographic, clinical, and pathological characteristics of the study cohort are summarized in **Supplementary Table 1**. The twelve patients who spared from receiving chemotherapy were all stage 1A, having low erythrocyte Sedimentation Rate (ESR), all having peripheral lymphadenopathy without bulky disease. While 6 of them having variant histology pattern (4 with pattern C and 2 with pattern E), 4/12 of those patients experienced relapse (median time to relapse was 29.93 months, ranging from 5.48-76.82 months). Two of the relapsing patients within this group were among variant histology patients, and all achieved CR after receiving R-CHOP without radiotherapy. The 5-year overall OS and EFS for this group were 100% and 71.4%, respectively. Among the 54 pediatric patients who received the R-CHOP regimen as primary treatment, 35 patients (64.8%) had an ESR less than 30 mm/hr, and 38 patients (70.4%) had less than three nodal sites involved initially. Fifty percent (27 patients) presented with stage 2 disease at presentation. Six patients (11%) had B symptoms and 48 patients (89%) did not have B symptoms. Bulky disease was present in 7 (13%) patients. Splenic and extra-nodal involvement were less common, occurring in 4 (7.4%) and 3 (5.6%) of the cases, respectively. Early-stage disease was encountered in 39 patients (72.2%) and 15 (27.8%) had advanced-stage disease. Variant histology pattern was observed in 33 (50%) of all studied cohort, 27 of them were among the group received R-CHOP initially. The criteria for patients who received R-CHOP as the initial line of treatment are presented in **Table 1**.

**Table 1:** The demographic, clinical, and pathological characteristics of the patients received RCHOP regimen as initial treatment.

Characteristics	Number (54)	% (100)
Peripheral lymphadenopathy		
Y N	42 12	77.8 22.2
Histological variants		
Y N	27 27	50 50
ESR		
<30 ≥30	35 19	64.8 35.2
Number of nodal involvement		
<3 ≥3	38 16	70.4 29.6
Staging		
1A 1B 2A 3A 3B 4A 4B	11 1 27 9 3 1 2	20.4 1.9 50 16.7 5.5 1.9 3.7
B symptoms		
Y N	6 48	11 89
Bulky disease		
Y N	7 47	13 87
Splenic involvement		
Y N	4 50	7.4 92.6
Extra-nodal disease		
Y N	3 51	5.6 94.4
Risk stratification		
Early stage Advanced stage	39 15	72.2 27.8
Number of RCHOP cycles		
3 4 6	9 30 15	16.6 55.5 27.7
Interim PET-CT		
Negative Positive	51 3	94.4 5.6
Evaluation at end of treatment		
CR by PET-CT CR by CT	26 28	48 52
Relapse		
Y N	6 48	11 89
Final outcome		
Alive Died	53 1	98 2

Y, yes; N, no; ESR, Erythrocyte Sedimentation Rate; RCHOP, (rituximab, cyclophosphamide, doxorubicin, vincristine and prednisone); PET-CT, positron emission tomography–computed tomography; CT, computed tomography; CR, complete response.

### 3.2 Measures of outcome and survival

**Figure 2:**
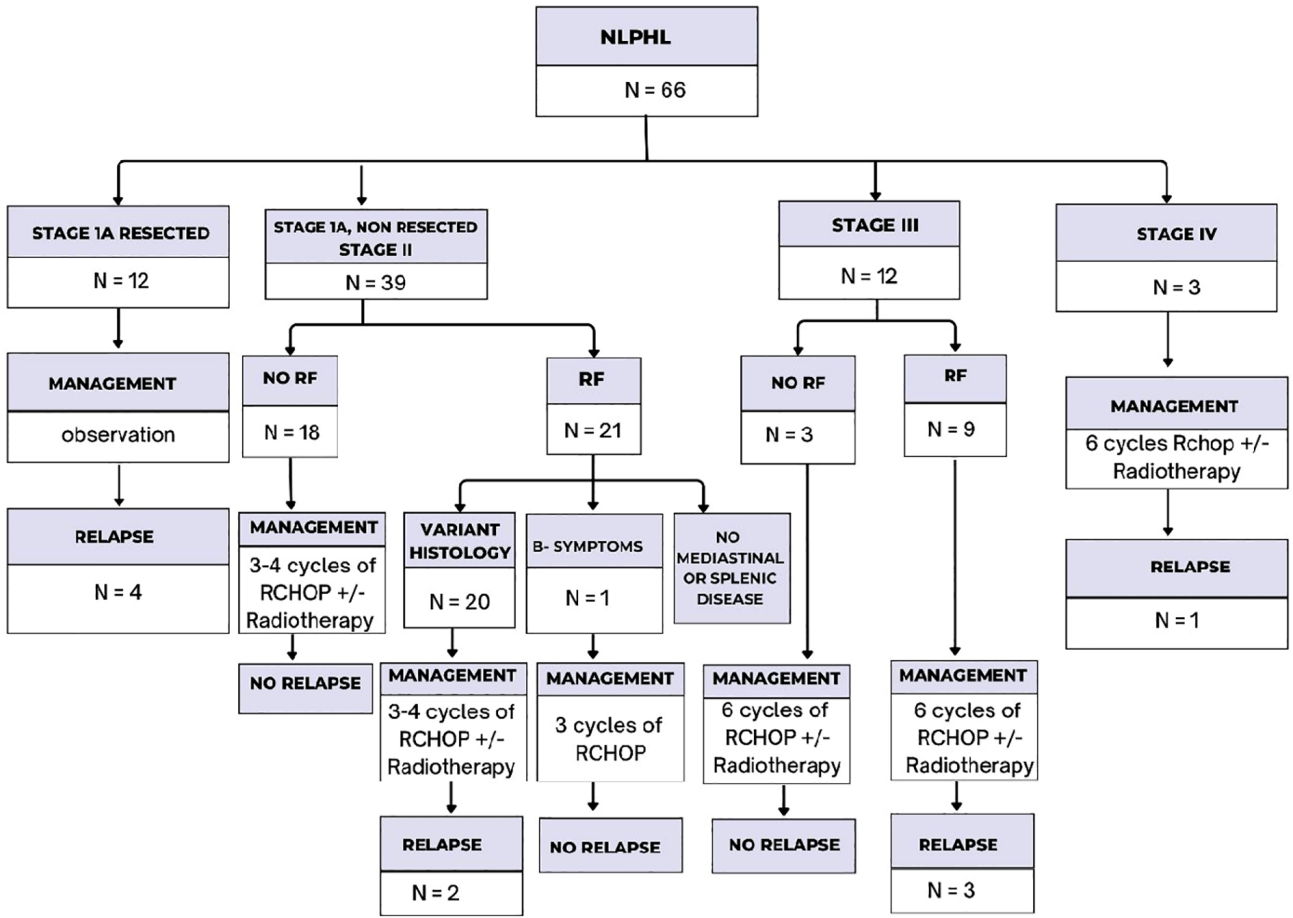
Management algorithm and outcomes of nodular lymphocyte predominant hodgkin lymphoma (N = 66). NLPHL, nodular lymphocyte predominant Hodgkin lymphoma; RF, risk factor.

**Figure 3:**
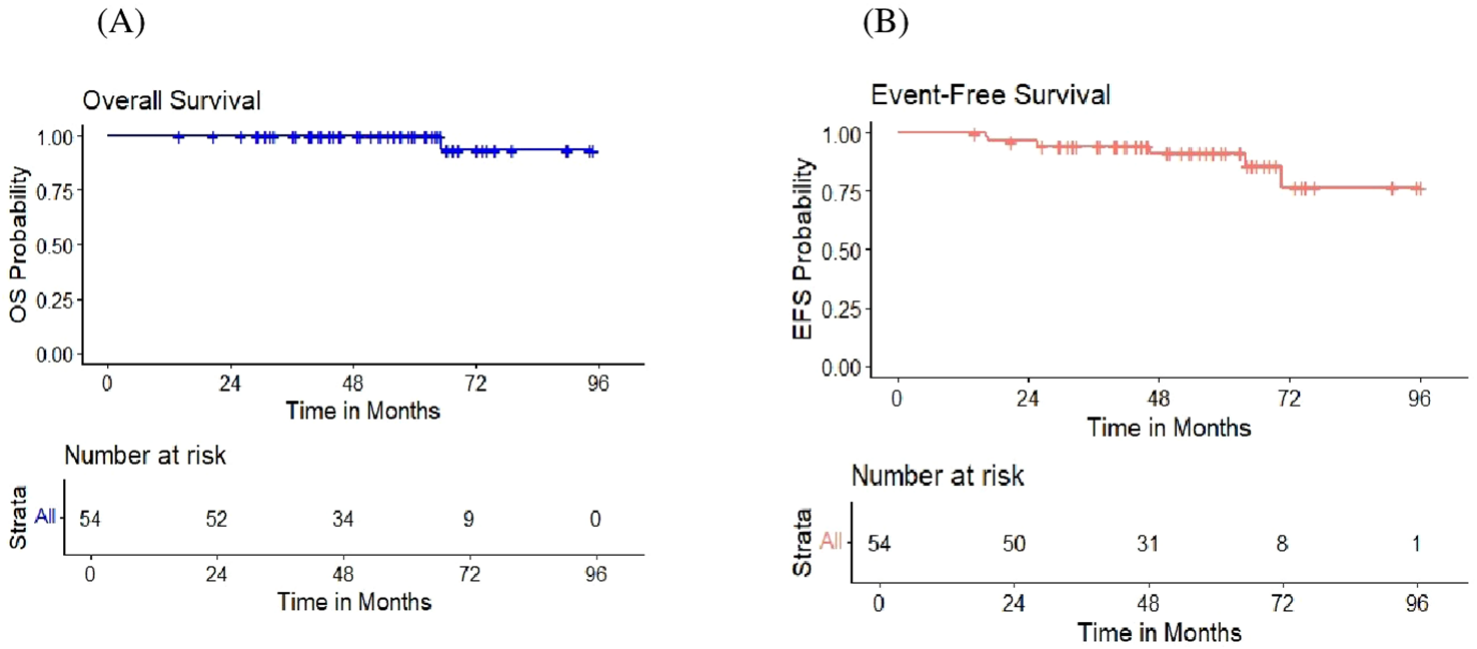
Survival curves for patients with nodular lymphocyte-predominant Hodgkin lymphoma (NLPHL) after R-CHOP. **(A)** The overall survival. **(B)** The event free survival.

After two cycles of R-CHOP, 51 patients (94.4%) achieved CR, demonstrating an excellent early treatment response, and the ORR to R-CHOP regimen as initial treatment was 100%. **Figure 2** highlights the detailed treatment algorithm and the outcomes of NLPHL patients after R-CHOP. Following R-CHOP, nine patients underwent consolidation radiotherapy, and this group did not experience any relapses. Relapse occurred in 11% of patients (6/54) who received R-CHOP as initial treatment during the follow-up period (median time to relapse was 37.97 months, ranging from 12.2-70.56). It was confirmed by a new tissue biopsy with no transformation observed. The majority of relapsing patients were successfully salvaged after an average of 4 to 6 cycles of R-ICE, with 5 patients achieving CR as evident by negative PET-CT following 2 cycles of R-ICE. They were then consolidated with ISRTH at a dose of 1980 cGy. Analysis using fisher’s exact test showed that among patients who received R-CHOP, those with B symptoms had a significantly higher incidences of relapse (p=0.024). In Patients with advanced-stage disease at presentation (n=15), 4 developed relapses compared to 2 patients in the early-stage disease group (n=39). Additionally, 5 of the 6 patients who relapsed were in the age category of more than 10 years. Interestingly, our results showed that there were no relapses observed among those with PR after the first 2 cycles of R-CHOP. The correlations between the different prognostic factors and the relapse risk are demonstrated in **Supplementary Table 2**. None of the relapsed patients received radiotherapy initially. Only one of the relapsed patients, underwent ASCT followed by consolidation radiotherapy because he did not achieve CR after 2 cycles of R-ICE, but relapsed afterward. This patient received 2 cycles each of R-DHAP and bendamustine/rituximab, and was scheduled for haploidentical bone marrow transplant (BMT), but unfortunately died in CR due to a measles infection. **Table 2** summarizes the details of the relapsed patients throughout the entire study, including relapse timing, disease stage, pathology, treatments, and outcomes. The median follow-up duration for patients who received R-CHOP as initial treatment was 56.52 months (range from 14.1-94.82). The 5-year OS for this group was 100%, as no deaths occurred within the first 5 years of follow-up. One death occurred after 5 years, at approximately 65 months (~6 years) from diagnosis, which does not affect the OS estimate at the 5-year timepoint but impacts OS beyond 5 years. The overall 5-year EFS for the same group was 91.5%, as shown in **Figure 3**.

**Table 2:** Details of the relapsed patients throughout the whole study, including relapse timing, disease stage, pathology, treatments, and outcomes.

No	Initial stage	Variant histology Y/N	Type of Variant histology	First line	Initial radiotherapy Y/N	Time of relapse	Stage at relapse	Second line	Response after 2 cycles of chemotherapy	Radiotherapy at relapse Y/N	BMT	Outcome
1	1A	Y	C	Observation	N	Late	3	6 RCHOP	CR	N	N	Alive in CR
2	3A	N	NA	6 RCHOP	N	Early	3	6 RICE	PR	Y	Y	Died in CR
3	1A	N	NA	Observation	N	Late	1	3 RCHOP	CR	N	N	Alive in CR
4	3B	N	NA	6 RCHOP	N	Late	3	6 RICE	CR	Y	N	Alive in CR
5	1A	Y	F	4 RCHOP	N	Late	2	6 RICE	CR	Y	N	Alive in CR
6	1A	Y	C	Observation	N	Early	2	4 RCHOP	CR	N	N	Alive in CR
7	1A	N	NA	Observation	N	Early	1	4 RCHOP	CR	N	N	Alive in CR
8	4B	Y	D	6 RCHOP	N	Late	3	6 RICE	CR	Y	N	Alive in CR
9	2A	Y	F	4 RCHOP	N	Late	2	4 RICE	CR	Y	N	Alive in CR
10	3A	Y	C	6 RCHOP	N	Late	3	6 RICE	CR	Y	N	Alive in CR

Y, yes; N, no; NA, not applicable; RCHOP, (rituximab, cyclophosphamide, doxorubicin, vincristine and prednisone); RICE, (Rituximab, Ifosfamide, carboplatin, and etoposide); CR, complete response; PR, partial response; BMT, bone marrow transplant.

**Figure 4:**
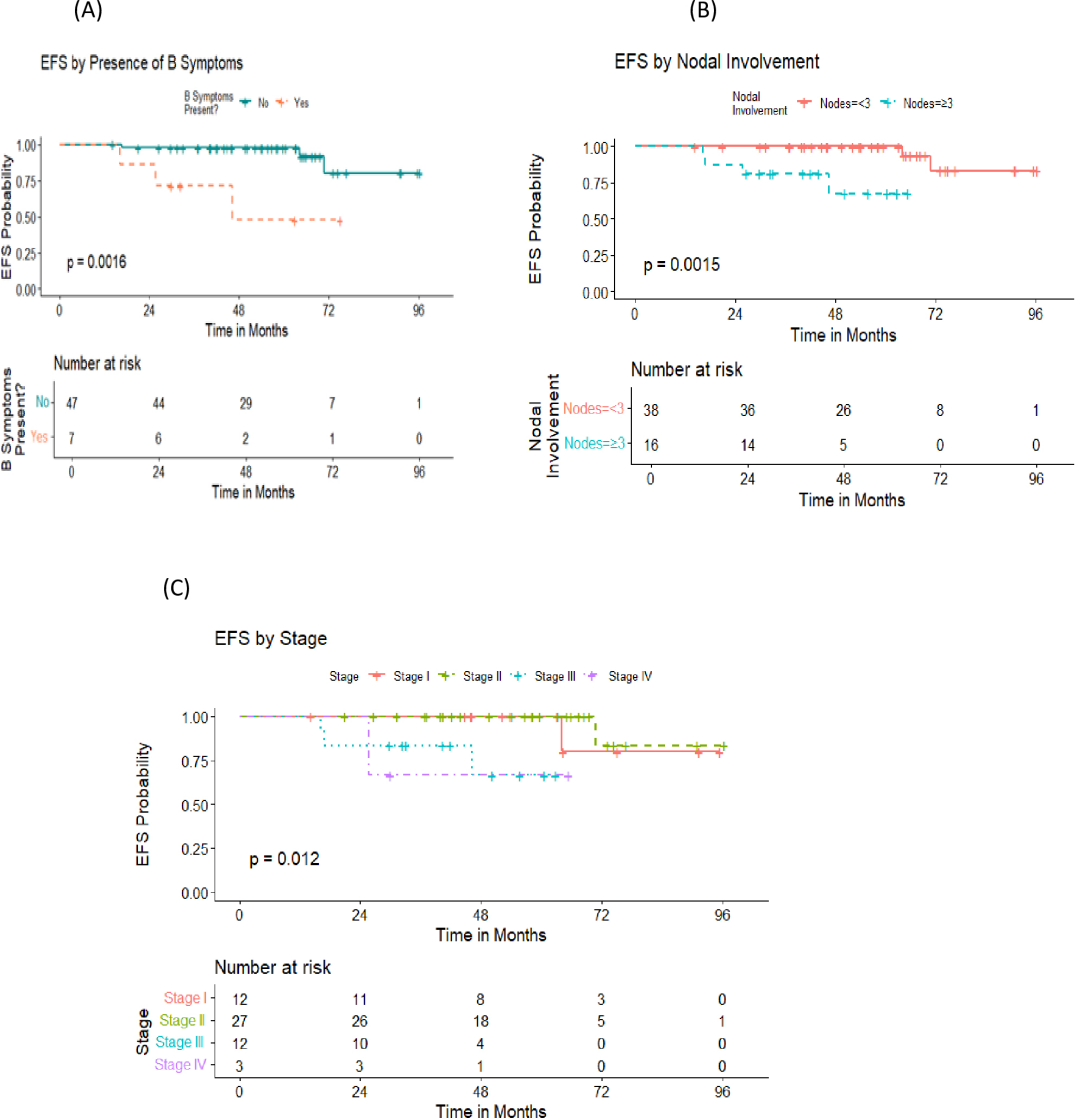
Outcome of the patients in relation to different prognostic factors. **(A)** Comparison of event free survival by the presence of B symptoms. **(B)** Comparison of event free survival by the number of nodal involvements. **(C)** Comparison of event free survival by disease stage.

**Figure 5:**
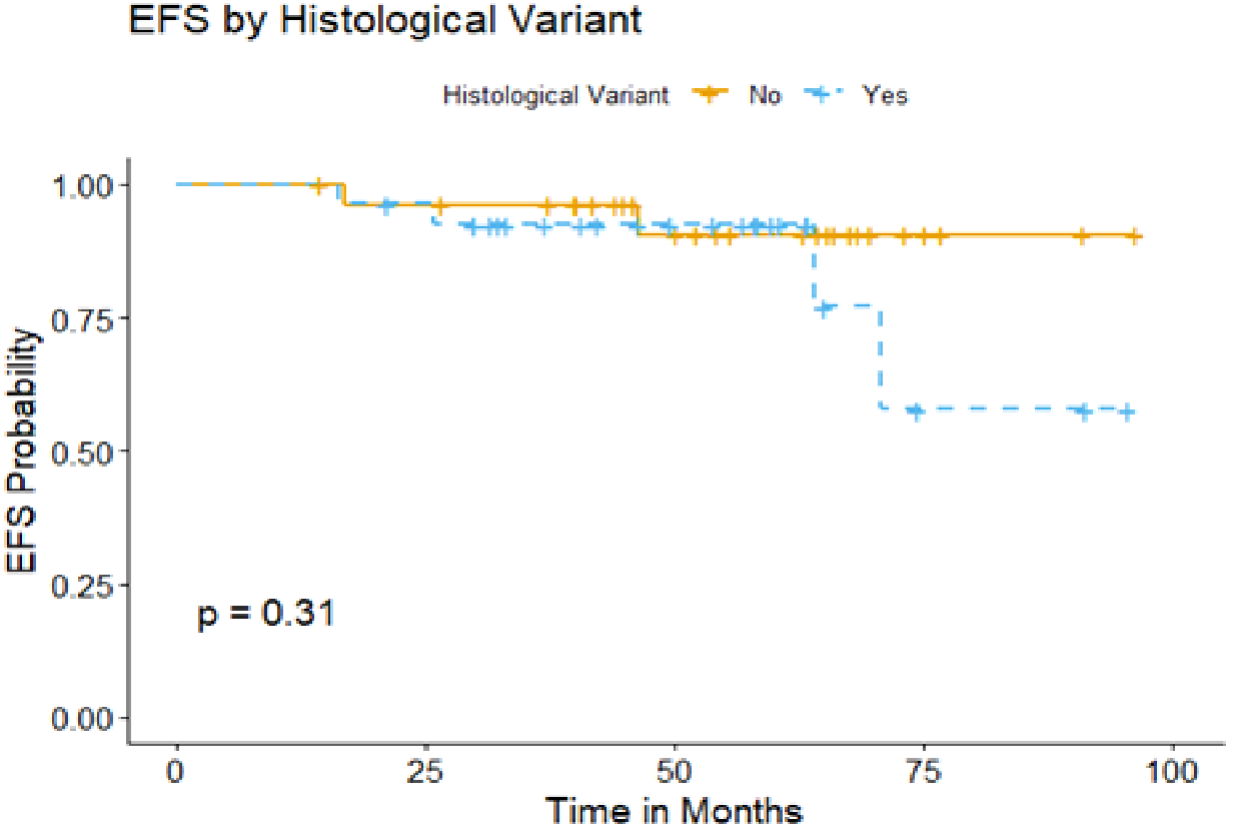
Comparison of the event free survival (EFS) of typical histology pattern versus variant histology pattern NLPHL among patients received R-CHOP.

**Figure 6:**
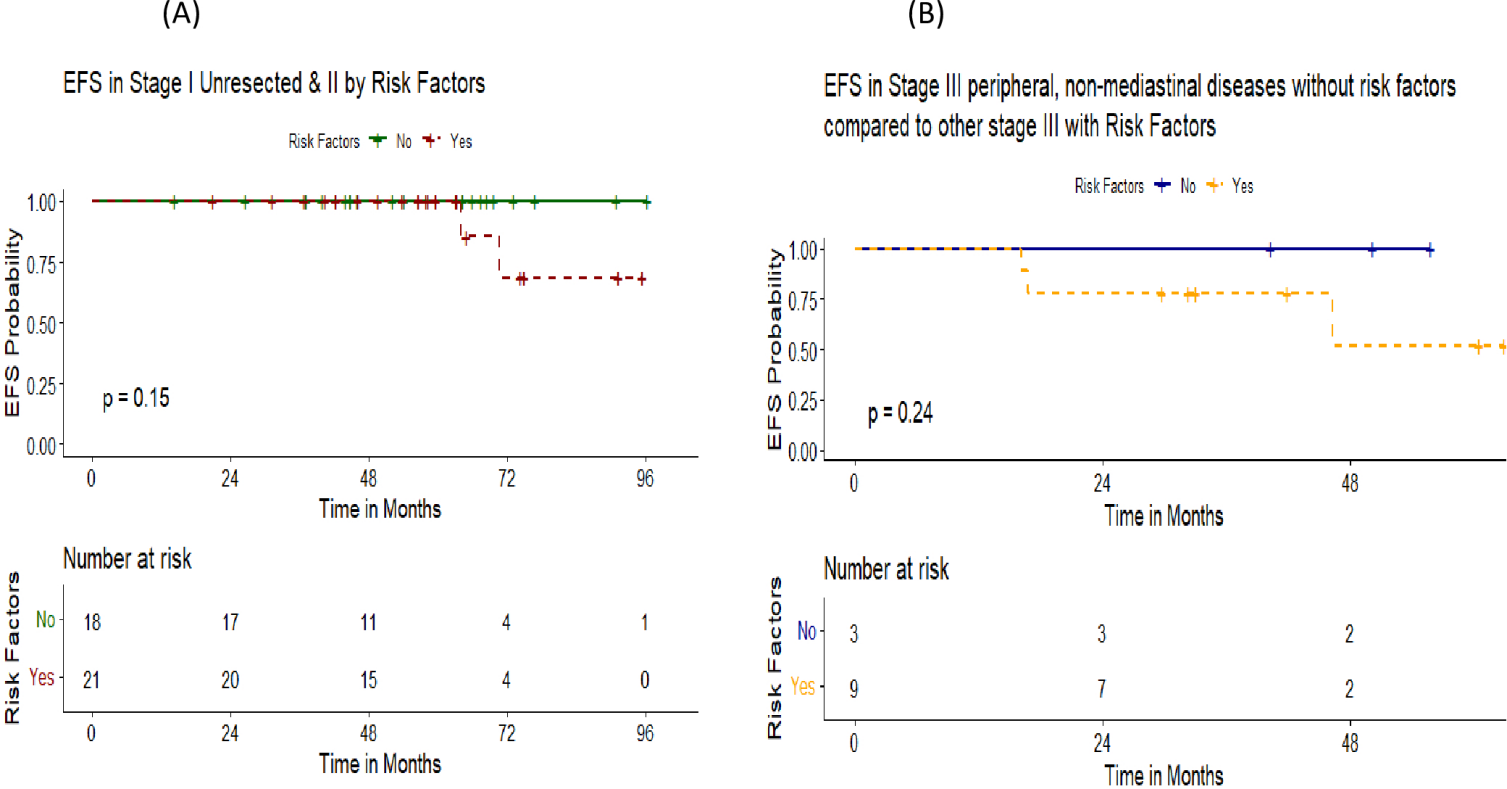
Event free survival stratified by disease stage and presence of risk factors. **(A)** Event free survival among patients with un-resected stage 1 and 2 by the presence of risk factors. **(B)** Event free survival among patients with stage 3 peripheral non-mediastinal disease without risk factors compared to other stage 3 with risk factors.

Those with early stage disease, no B symptoms initially and involvement of less than 3 nodal sites had significantly higher 5-year EFS compared to advanced stage, B symptoms and more than 3 nodal sites affection (100% vs 68.6%, 97.8% vs 47.6% and 100% vs 67.7%, respectively) as demonstrated in **Figure 4**. Notably, the presence of variant histological pattern among patients who received R-CHOP regimen did not significantly impact the 5 -year EFS (**Figure 5**). In our analysis, patients with non-resected stage 1 or stage 2 disease without risk factors remained relapse-free, achieving a 5-year EFS of 100%. In contrast, events occurred exclusively among patients with any risk factor within the same stages, resulting in a decline of EFS to 68.6% at 72 months. In a similar vein, patients with stage 3 disease without any risk factors demonstrated an excellent 5-year EFS of 100%, whereas those with at least one risk factor showed a marked decline in EFS to 51.9% as in **Figure 6**.

### 3.3 Toxicity

Out of the 254 administered R-CHOP cycles, grade 3 neutropenia was observed in 9% (23/254) and grade 4 neutropenia in 13% (33/254) of the cycles. Febrile neutropenia requiring hospital admission occurred in 4% of the cycles, with a hospital stay ranging from 4 to 7 days; no blood stream infections were documented. One patient developed appendicitis and underwent an appendectomy in the absence of neutropenia. Another patient required dose reductions of doxorubicin and cyclophosphamide due to recurrent myelo-suppression.

## 4 Discussion

Unlike cHL, NLPHL has no clearly established definitive treatment protocol due to rarity of the disease. Given the heterogonous types of treatment modalities used for this disease, there is a need to fill the gap in the current literature about the best treatment option that can reduce the risk of relapse and transformation. Traditionally, NLPHL has been treated using therapeutic strategies designed for cHL. However, this approach remains controversial, as the distinct biological characteristics and typically indolent clinical course of NLPHL may lead to overtreatment and increased toxicity when managed with conventional cHL-based regimens (15, 16).

Our cohort aimed to assess the outcome and impact of different prognostic factors among patients with NLPHL who received a unified chemotherapy regimen.

Numerous studies have reported patients with early stage NLPHL, including those with completely resected stage 1A, to have excellent long-term survival (17, 18). So, there is a trend towards de-escalation of treatment for this highly selected subgroup in order to reduce the adverse events associated with chemotherapy and radiotherapy without compromising the prognosis. The Children’s Oncology Group (COG) evaluated a surgery-only strategy in children and young adults with stage IA NLPHL who had no residual disease after lymph node resection in a prospective, non-randomized trial called AHOD03P1 (17). 52 patients had complete resection, as determined by PET-CT scan; as a result, they were spared radiation and chemotherapy. Thirteen of these patients experienced a recurrence of their disease, which was successfully treated with CHOP (doxorubicin/vincristine/prednisone/cyclophosphamide). This group had a 5-year EFS of 77%.

Also, in the Euro Net-PHL study (19), children with fully excised stage IA disease who did not receive any additional upfront treatment had a 5-year OS of 100% and PFS of 67%. This is consistent with our reported 71% EFS for this particular group, and 4–6 cycles of R-CHOP were effective in saving all relapsed patients in our cohort (4/12).

Patients with non-resected early-stage NLPHL (stage I/II) can still have excellent outcome with minimal chemotherapy doses. The COG study AHOD03P1 (17), which enrolled 135 children with low-risk, non-bulky stage IA or IIA non-resected NLPHL, a de-escalated chemotherapy strategy (three cycles of CHOP) was tested to minimize therapy while preserving excellent outcomes. The reported 5-year EFS in this cohort was approximately 85.5%, and OS was 100%.

Another treatment option might be the CVP regimen, which consists of vinblastine, prednisolone, and cyclophosphamide. The CR rate was 80% in an Anglo-French collaborative report (20) that included 45 pediatric patients with early-stage NLPHL who received three cycles of CVP as first-line therapy. The freedom-from-treatment-failure (FFTF) rate was 75.4%, with 12 patients experiencing an event after a median follow-up of 40 months. There were no acute toxicities reported, and the OS rate was 100%.

Furthermore, the German Hodgkin Study group (GHSG) recommends the CVP regimen for patients with early-stage disease (21). The GHSG evaluated 256 adult patients with stage IA NLPHL in a large retrospective analysis; 108 of these patients received involved-field radiation therapy (IFRT) alone, and their 8-year PFS and OS rates were 91.9% and 99%, respectively. In 17 patients (6.6%), secondary malignancies were discovered during follow-up (22). Because of the concerns about late toxicities in a disease with a prolonged survival, pediatric cooperative groups typically aim to minimize or avoid the use IFRT alone, unlike adults, where it has been used successfully. The clinical utility of rituximab in NLPHL is still up for debate, with evidence supporting improved PFS when combined with upfront chemotherapy, contrasted by poor results with single-agent use (5, 23, 24). Patients with unresectable stage I or II disease without unfavorable features in our cohort did not relapse after three to four cycles of R-CHOP. Given the favorable results in this subgroup, a brief course of low-intensive chemotherapy, like R-CVP (3 cycles), might be a desirable option of treatment with a lower chance of side effects linked to late-onset treatment. Advanced-stage NLPHL is uncommon. Unlike early-stage disease, it often demonstrates more aggressive biology, poorer treatment response, and a higher risk of relapse with no consensus exists on the optimal therapeutic approach (25). The outcomes of patients with stage IIIA NLPHL who present with non-mediastinal peripheral nodal involvement, such as cervical, axillary, external iliac, or inguino-femoral lymphadenopathy, and who do not have unfavorable characteristics, such as splenic involvement or variant histology, are often similar to those of patients with stage IIA disease.

Additionally, the majority of relapsed cases in this group can be successfully treated with additional low-intensity chemotherapy (26–28). This is comparable with our findings, showing excellent EFS with no relapses in the 3 patients of this subgroup after 6 cycles of R-CHOP, suggesting that less intensive regimens like R-CVP represents a reasonable treatment option for these patients (29). However, a larger sample size is needed to reach more reliable conclusions.

In contrast, high risk advanced stage disease, including stage 3 with risk factor and stage 4, are more aggressive and necessitate more intensive treatment. The Anglo-French report on children and adolescents with advanced-stage NLPHL demonstrated that both B-NHL and cHL chemotherapy regimens achieve comparable outcomes and recommends rituximab in combination with multi-agent alkylator-containing chemotherapy, such as R-CHOP, as first-line treatment (25).

Relapse risk in our cohort was 33.3% (4/12) in this high-risk group, indicating that additional chemotherapy intensification, up to six cycles of R-CHOP, might be necessary. The role of ABVD in the treatment of NLPHL remains a subject of debate. In early stage disease, short course ABVD followed by ISRTH has achieved favorable outcomes, with reported 10-year PFS of approximately 80% and OS exceeding 90% in retrospective studies. In contrast, ABVD alone has shown inferior disease control in advanced-stage NLPHL, largely due to the distinct biological behavior of this subtype (). In a matched-pair analysis comparing 42 patients with advanced NLPHL and 84 21with advanced cHL, most treated with ABVD or ABVD-like regimens, time to progression was significantly shorter among NLPHL patients, primarily due to higher relapse rates and an increased risk of transformation to aggressive B-cell NHL (1). According to a study conducted at our center, ABVD was linked to a significantly higher risk of relapse (17/37) than the group that received R-CHOP (0/16), which resulted in worse disease control. The 3-year EFS for the ABVD group was 65%, whereas the R-CHOP group’s was 100%, P = 0.04 (8).

Variant histology has consistently been identified as an independent adverse prognostic factor, associated with advanced-stage presentation, inferior treatment outcomes, and an increased risk of recurrence in both pediatric and adult populations. The prognostic significance of growth patterns (GP), especially when categorized as GP AB versus GP CDEF, has been shown in a number of studies. In a GHSG analysis of 413 patients with NLPHL who were enrolled in prospective trials, patients with GP AB had a significantly higher 5-year PFS than those with GP CDEF patterns (12, 30). In our group, 7 out of 12 patients with stage IA disease who had total resection and were monitored showed variant histology. The relapse pattern in these patients did not differ from those with typical histology, suggesting that observation may remain an appropriate management strategy for completely resected stage IA cases with variant histology. Nevertheless, confirmation through larger studies is warranted.

Among patients with un resected stage I or II disease, variant histology was observed in 51% (20/39), with only two relapses occurring after 3–4 cycles of R-CHOP. In the current study, variant histology did not significantly affect 5-year EFS among patients treated with R-CHOP, including those in early stages who received fewer treatment cycles. In the same way, in advanced-stage disease, relapse rates were comparable between patients with and without variant histology (two cases in each group). However, a prior study conducted at our center by Ali et al. (6) found that patients with variant histology who received ABVD (33.3%) had a significantly lower EFS than those who received R-CHOP (94.4%; p = 0.01). Notably, among patients treated with R-CHOP, variant histology did not adversely influence outcomes, with 5-year EFS rates remaining nearly identical between groups (approximately 95%). These findings suggest that R-CHOP may mitigate the unfavorable prognostic effect of variant histology and offer superior disease control compared with ABVD-based therapy.

The 5-year OS and EFS rates for our entire cohort were 100% and 91.5%, respectively. These findings are consistent with a study conducted at MD Anderson Cancer Center (10), which reported a 5-year PFS of 91.3% among patients treated with R-CHOP after excluding those with histological transformation.

Although PET-CT is a well-established tool for early response assessment in cHL, its predictive utility in NLPHL remains uncertain, largely due to the distinct biological behavior and metabolic FDG uptake patterns of NLPHL compared with cHL (31). In our series, all three patients who were PET-positive after two cycles of R-CHOP remained in remission, whereas, relapse occurred exclusively among those with a negative interim PET-CT. In contrast, Madney et al. found that relapse risk was significantly correlated with early PET-CT response after two cycles of R-CHOP (8). The primary checkpoint for assessing treatment response and forecasting results in NLPHL is the end-of-treatment assessment, as opposed to cHL. The COG study AHOD03P1 demonstrated that end of treatment PET-CT following three cycles of CHOP was predictive of relapse risk in low risk NLPHL, with a 5-year EFS of 90% for PET-negative patients compared to 66.7% for PET-positive patients (17).

In our cohort, all patients achieved CR at the end of therapy, which may explain the favorable treatment outcomes observed.

A subset of patients with NLPHL experience relapse, sometimes multiple and frequently late in the course of the disease, despite the fact that the majority have a favorable overall prognosis (32). Management of relapsed NLPHL remains poorly defined, and the role of high-dose chemotherapy (HDC) followed by ASCT as salvage therapy continues to be debated (33). The GHSG conducted a retrospective analysis of 99 patients with relapsed or refractory NLPHL enrolled across 12 prospective trials, reporting 5-year PFS and OS rates of 76% and 90%, respectively, following salvage therapy for first relapse or progression. Outcomes were comparable across various treatment approaches, suggesting that while treatment intensification may be appropriate for selected high risk patients, a more conservative approach is often sufficient (34). In our cohort, 5 of the 6 relapsed patients post R-CHOP achieved CR following salvage chemotherapy combined with radiotherapy and remained in remission and alive. Only one patient had PR after 2 cycles of salvage therapy and proceeded to ASCT; but relapsed later on, and died in CR due to measles infection. These findings support the notion that ASCT may not be routinely indicated for first relapse when a good response to salvage therapy is achieved and can be reserved for patients with multiple relapses or refractory disease. This further underscores the distinct biological and clinical behavior of NLPHL compared with cHL in children, reinforcing the need for tailored, risk-adapted management strategies.

NLPHL is associated with a recognized, though variable, risk of histologic transformation into aggressive B-cell lymphoma, most frequently DLBCL. While no cases of transformation were observed in our cohort, this possibility cannot be excluded and may become evident with longer follow-up. Previous studies have reported variable rates of transformation, underscoring the importance of extended surveillance. The British Columbia Cancer Agency study reported transformation rates of 7% at 10 years and 30% at 20 years among 95 NLPHL patients (median follow-up ~6.5 years) (35). Similarly, long-term data from the Mayo Clinic, encompassing 222 patients with a median follow-up of approximately 16 years, demonstrated a transformation rate of 7.6% and an annual risk of 0.74 per 100 patient-years (36). The absence of transformation in our series may, in part, be attributed to the use of the R-CHOP regimen, which incorporates anti-CD20 therapy, a key component of standard DLBCL treatment and a potential factor in reducing the risk of transformation.

Moreover, splenic involvement at diagnosis has been identified as a major risk factor for histologic transformation (35–37). In our cohort, splenic involvement was documented in only 7.4% of patients, which may have further contributed to the low observed risk. Collectively, these findings highlight the potential protective role of anti-CD20 based regimens and reinforce the need for long-term follow-up to better define the true risk of transformation in pediatric NLPHL.

While our study successfully identified significant predictors through univariate testing, a key limitation is the inability to perform a subsequent multivariate analysis to confirm their independence. This will frame our findings as identifying crucial risk factors that need further investigation in larger, multi-center studies, which could support the development of more refined risk-stratification models.

## 5 Conclusion

R-CHOP demonstrated excellent tolerability and promising efficacy across all stages of pediatric NLPHL. Patients with early stage disease achieved particularly favorable outcomes, supporting consideration of treatment de-escalation in this subgroup. Furthermore, R-CHOP appeared to mitigate adverse prognostic factors, including variant histology. Relapsed NLPHL cases were effectively managed with salvage chemotherapy, with or without radiotherapy, allowing autologous transplantation to be reserved for those with a second relapse. Given its indolent nature and distinct biological behavior, NLPHL warrants a management approach distinct from cHL. Developing standardized, evidence-based treatment protocols tailored to this rare entity is crucial to further optimize outcomes in the pediatric population.

## Data Availability

The datasets presented in this study can be found in online repositories. The names of the repository/repositories and accession number(s) can be found in the article/**Supplementary Material.**
