# Supplementary material for: Promising outcomes of R-CHOP therapy in pediatric nodular lymphocyte-predominant Hodgkin lymphoma: perspectives from a rare subtype
Source: Front Oncol. 2026 Mar 16;16:1740063. doi: 10.3389/fonc.2026.1740063 (PMC13033539; doi:10.3389/fonc.2026.1740063)
Supplement: Supplementary file 1 [file Table1.docx]

**Supplementary table 1** The demographic, clinical, and pathological characteristics of the whole study cohort.

| **Characteristics** | **Number** | **%** |
| --- | --- | --- |
| **Sex**  M  F | 50  16 | 75.8  24.2 |
| **Age**  1-5  >5-10  >10 | 6  25  35 | 9.1  37.9  53 |
| **ESR**  <30  ≥30 | 46  20 | 69.7  30.3 |
| **Mediastinal lymphadenopathy**  Y  N | 7  59 | 10.6  89.4 |
| **Stage at presentation**  I  II  III  IV | 24  27  12  3 | 36.4  40.9  18.1  4.5 |
| **B symptoms**  Y  N | 6  60 | 9  91 |
| **Histological pattern**  Typical histology pattern  Variant histology pattern | 33  33 | 50  50 |
| **Splenic involvement**  Y  N | 4  62 | 6.1  93.9 |
| **Bulky disease**  Y  N | 7  59 | 10.6  89.4 |
| **Location of lymphadenopathy**  Supra-diaphragmatic  Infra-diaphragmatic  Supra and infra-diaphragmatic | 41  11  14 | 62.1  16.7  21.2 |
| **Initial PET CT**  Positive  Negative | 54  12 | 81.8  18.2 |

M, male; F, female; Y, yes; N, no; PET CT, positron emission tomography–computed tomography

**Supplementary table 2** Prognostic Factors influencing the relapse risk in children with nodular lymphocyte-predominant Hodgkin lymphoma received RCHOP as initial treatment.

| **Characteristic** | **Relapse: No (N=48)** | **Relapse: Yes (N=6)** | ***P*-Value***¹* |
| --- | --- | --- | --- |
| **B symptoms** |  |  | **0.024*** |
| No | 44 (91.7%) | 3 (50.0%) |  |
| Yes | 4 (8.3%) | 3 (50.0%) |  |
| **Stage at presentation** |  |  | 0.091 |
| Stage I-II | 37 (77.1%) | 2 (33.3%) |  |
| Stage III-IV | 11 (22.9%) | 4 (66.7%) |  |
| **ESR category** |  |  | 0.123 |
| Less than 30 | 32 (66.7%) | 3 (50.0%) |  |
| Greater than or equal to 30 | 16 (33.3%) | 3 (50.0%) |  |
| **Age category** |  |  | 0.182 |
| 1 to 5 years | 4 (8.3%) | 1 (16.7%) |  |
| More than 5 to 10 years | 18 (37.5%) | 0 (0.0%) |  |
| More than 10 years | 26 (54.2%) | 5 (83.3%) |  |
| **Extranodal disease** |  |  | 0.303 |
| No | 46 (95.8%) | 5 (83.3%) |  |
| Yes | 2 (4.2%) | 1 (16.7%) |  |
| **Splenic involvement** |  |  | 0.385 |
| No | 45 (93.8%) | 5 (83.3%) |  |
| Yes | 3 (6.2%) | 1 (16.7%) |  |
| **Interim PET** |  |  | 1.000 |
| Negative | 45 (93.8%) | 6 (100.0%) |  |
| Positive | 3 (6.2%) | 0 (0.0%) |  |
| **Bulky disease (> 6 cm)** |  |  | 1.000 |
| No | 41 (85.4%) | 6 (100.0%) |  |
| Yes | 7 (14.6%) | 0 (0.0%) |  |
| **Response (CR/PR)** |  |  | 1.000 |
| CR | 45 (93.8%) | 6 (100.0%) |  |
| PR | 3 (6.2%) | 0 (0.0%) |  |

ESR, Erythrocyte Sedimentation Rate; PET CT, positron emission tomography–computed tomography; CR, complete response; PR, partial response
